# Comparison of the Performance of GPT-3.5 and GPT-4 With That of Medical Students on the Written German Medical Licensing Examination: Observational Study

**DOI:** 10.2196/50965

**Published:** 2024-02-08

**Authors:** Annika Meyer, Janik Riese, Thomas Streichert

**Affiliations:** 1 Institute for Clinical Chemistry University Hospital Cologne Cologne Germany; 2 Department of General Surgery, Visceral, Thoracic and Vascular Surgery University Hospital Greifswald Greifswald Germany

**Keywords:** ChatGPT, artificial intelligence, large language model, medical exams, medical examinations, medical education, LLM, public trust, trust, medical accuracy, licensing exam, licensing examination, improvement, patient care, general population, licensure examination

## Abstract

**Background:**

The potential of artificial intelligence (AI)–based large language models, such as ChatGPT, has gained significant attention in the medical field. This enthusiasm is driven not only by recent breakthroughs and improved accessibility, but also by the prospect of democratizing medical knowledge and promoting equitable health care. However, the performance of ChatGPT is substantially influenced by the input language, and given the growing public trust in this AI tool compared to that in traditional sources of information, investigating its medical accuracy across different languages is of particular importance.

**Objective:**

This study aimed to compare the performance of GPT-3.5 and GPT-4 with that of medical students on the written German medical licensing examination.

**Methods:**

To assess GPT-3.5’s and GPT-4's medical proficiency, we used 937 original multiple-choice questions from 3 written German medical licensing examinations in October 2021, April 2022, and October 2022.

**Results:**

GPT-4 achieved an average score of 85% and ranked in the 92.8th, 99.5th, and 92.6th percentiles among medical students who took the same examinations in October 2021, April 2022, and October 2022, respectively. This represents a substantial improvement of 27% compared to GPT-3.5, which only passed 1 out of the 3 examinations. While GPT-3.5 performed well in psychiatry questions, GPT-4 exhibited strengths in internal medicine and surgery but showed weakness in academic research.

**Conclusions:**

The study results highlight ChatGPT’s remarkable improvement from moderate (GPT-3.5) to high competency (GPT-4) in answering medical licensing examination questions in German. While GPT-4’s predecessor (GPT-3.5) was imprecise and inconsistent, it demonstrates considerable potential to improve medical education and patient care, provided that medically trained users critically evaluate its results. As the replacement of search engines by AI tools seems possible in the future, further studies with nonprofessional questions are needed to assess the safety and accuracy of ChatGPT for the general population.

## Introduction

Rapid advancements in large language models (LLMs) have sparked considerable excitement regarding their potential applications in the medical field [1,2]. One LLM-based application that has garnered worldwide attention is ChatGPT, developed by the research and deployment company OpenAI, due to its easy accessibility and potential to democratize knowledge [3]. The freely available version is based on the artificial intelligence (AI)–based tool GPT-3.5, which encompasses billions of parameters and has been trained on approximately 570 GB of text from the internet [1,2].

ChatGPT’s GPT-3.5 iteration has already shown promise in several routine medical tasks and medical research [4-7], even raising ethical concerns in the literature [2,3,8]. The prompt and interactive nature of this AI’s responses might even revolutionize search engines, while also revealing shortcomings in medical education [9-11]. However, despite the introduction of the more advanced iteration GPT-4, concerns about the lack of transparency regarding this AI’s model parameters, training process, and underlying data structure remain unaddressed [8,12]. These concerns cast doubt on the medical proficiency of these LLMs, as both were not primarily trained on medical data and are the first to admit that as a language AI model, passing a medical examination is outside their skillset (Multimedia Appendix 1). Still, with assistance and adaptations, GPT-3.5 nearly passed the United States Medical Licensing Examination [13,14], and GPT-4 passed a Japanese medical examination [15]. Considering the variable performance of multilingual LLMs across different input languages [16,17], it is imperative to evaluate these models in various other linguistic contexts as well as on large data sets of original medical examination questions.

The primary objective of this study is to evaluate the medical proficiency of both ChatGPT iterations (GPT-3.5 and -4) in comparison to medical students by testing it on 937 original questions from the written German medical licensing examination (Zweites Staatsexamen), providing further data for a possible future integration. While the German medical licensing examination covers various medical subdisciplines in 320 multiple-choice questions [18], it has a high interexamination reliability of over 0.9 [19]. Despite using the same third-party client for question retrieval as earlier studies, the German approach of publicly releasing the examination questions enables the third-party client to guarantee the originality of the test items derived directly from the examination itself [20]. Additionally, to the best of our knowledge, we have tested both ChatGPT versions on the largest data set of medical licensing examination questions not included in their training data set. Furthermore, we did not exclude all image-based questions a priori. Instead, we evaluated the relevance of the images for each question and compared the results both with and without images.

## Methods

### Data Collection

**Figure 1:**
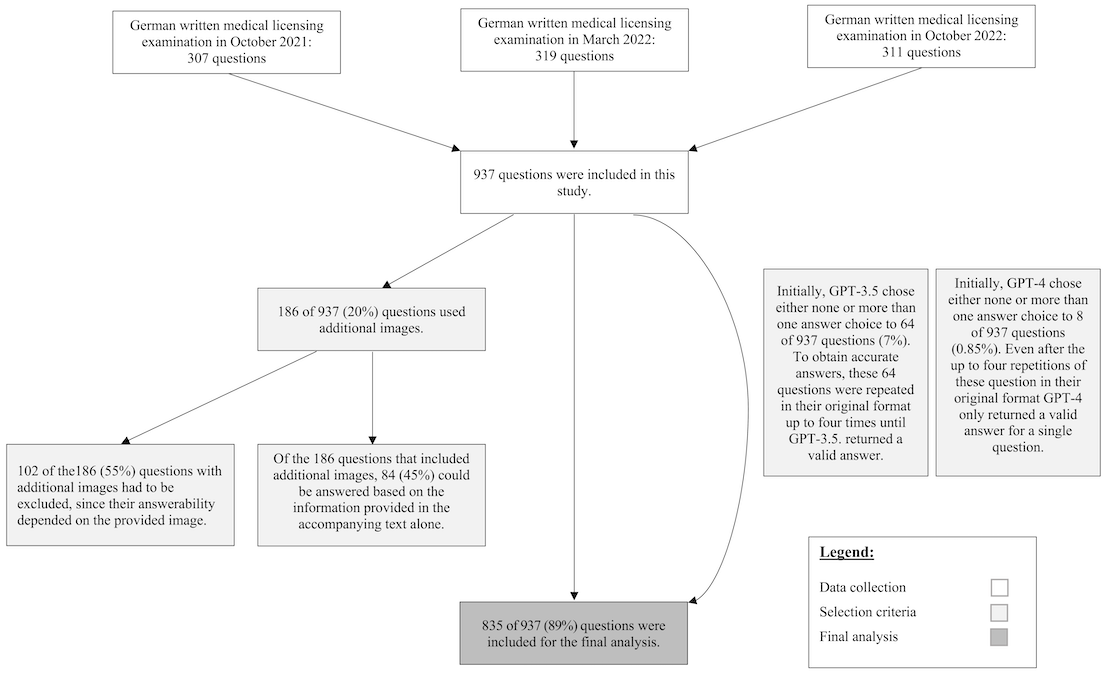
Flowchart of the study design for the evaluation of ChatGPT’s (GPT-3.5 and GPT-3) accuracy in the written German medical licensing examination (2021-2022). The flowchart presents the criteria for question selection, including both the inclusion and exclusion criteria.

To ensure that any observed performance was not influenced by changes in ChatGPT’s training data, we specifically chose the 3 most recent examinations (October 2021, April 2022, and October 2022) after the AI’s knowledge cutoff date [17]. Thus, we were able to obtain 937 multiple-choice questions, each with 5 possible answers from the third-party client Amboss, a web-based learning platform that provides the original questions from the Institut für Medizinische und Pharmazeutische Prüfungsfragen (IMPP). To maintain the original examination format, we presented all obtained questions and answer options in the same order as they appeared in the examination. No specific training code was used while submitting the questions. Due to AI’s inability to analyze visual content, answerability based on question text alone was defined as the primary inclusion criterion, resulting in the exclusion of 102 questions. The questions were submitted through ChatGPT’s interface of the GPT-3.5 (January 30, 2023) and GPT-4 (March 14, 2023) versions. ChatGPT’s answers were then compared to the official correct answers and evaluated. If ChatGPT selected none or more than 1 of the multiple-choice answers, the question was repeated in its original format up to 4 times or until a conclusive response could be obtained from ChatGPT (Figure 1).

We recorded additional data, such as answer length, content warnings, and recommendations for further diagnosis, and categorized the questioning methodology. To assess the readability of a question, we used the Simple Measure of Gobbledygook (SMOG) as it has shown acceptable interrater reliability for patient education materials in the literature [21].

Examination statistics provided by the “MEDI-LEARN” portal were also used, including the number of correct student answers and the specialization of each question. The “Blueprint” published by the IMPP outlines the distribution of subspecialties within the written state examinations [18].

### Statistical Analysis

To perform our data analysis, we used several packages [22-37] in addition to the R programming language [38].

While continuous variables were reported as arithmetic mean (SD) values, categorical variables were reported as frequencies and percentages. The Kolmogorov-Smirnov test, Shapiro-Wilk test, and QQ plots were used to confirm the normal distribution of continuous data statistically and graphically. To determine significant differences, we used unpaired *t* test or ANOVA for continuous variables and chi-square test or Wilcoxon rank-sum test for categorical variables. *P* values of <.05 were deemed significant. Univariate and multivariate regression analyses were additionally performed to provide information on probabilities and predictors.

### Ethical Considerations

Ethics approval was not required as data were collected from publicly available sources on the internet or were generated using AI-based methods. No personally identifiable information was used in the data collection, and all data were handled in accordance with applicable data privacy laws and regulations.

## Results

**Figure 2:**
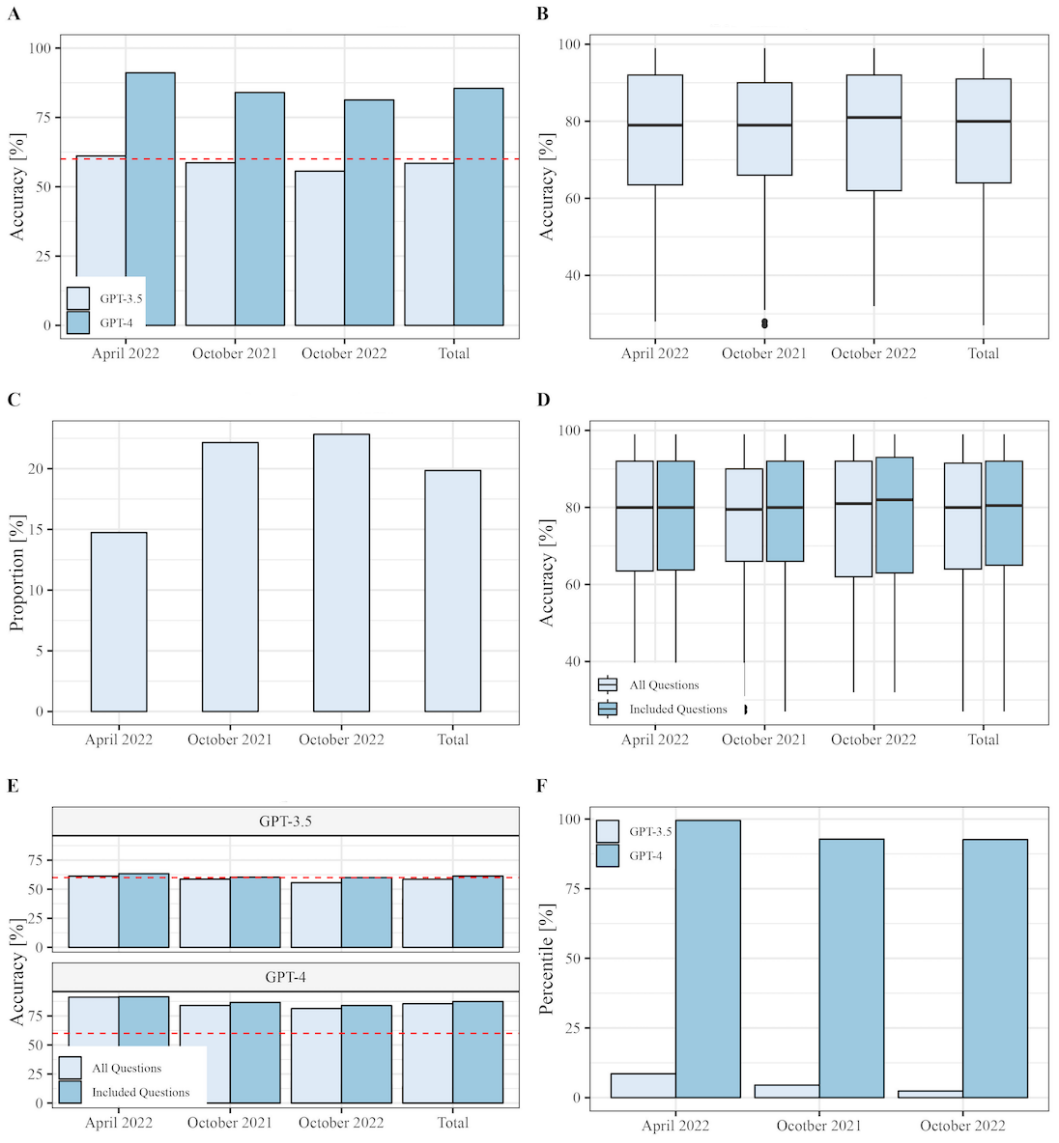
Bar plots of ChatGPT’s (GPT-3.5 and GPT-4) and box plots of students’ accuracy in the written German medical licensing examination (2021-2022). Bar graphs and box plots of (A) the relative number of correct answers provided by ChatGPT (GPT-3.5 and GPT-4) answers, (B) correct answers provided by students, (C) and image-based questions for the different examinations. (D and E) The relative number of correct answers by ChatGPT (GPT-3.5 and GPT-4) and students, comparing all questions with the included text-based questions. The 60% pass mark is presented as a red line in (A) and (E) to provide context for the performance of ChatGPT (GPT-3.5 and GPT-4). In addition, (E) displays the percentile achieved by ChatGPT (GPT-3.5 and GPT-4) for each year's examination, based on the percentile limits published by the Institut für Medizinische und Pharmazeutische Prüfungsfragen [37-39].

Overall, GPT-4 demonstrated superior performance with an average score of 796 out of 937 (85%), surpassing GPT-3.5’s score of 548 out of 937 (58%), which previously fell below the general passing threshold of 60% (Figure 2A) [37-39]. For the April 2022 examination, GPT-3.5 and GPT-4 achieved their highest scores (GPT-3.5: 195/319, 61%; GPT-4: 287/315, 91%), while the proportion of students who answered correctly remained constant across the 3 examinations (mean 76%, SD 18%; *P*=.86; Figure 2B and Multimedia Appendix 2).

Thus, GPT-4 passed all tested examinations, whereas GPT-3.5 could only pass 1 of the 3 examinations. Although the examinations varied in several aspects, we also observed a significant difference in the number of images (*P*=.02; Figure 2C and Multimedia Appendix 2). As GPT-3.5 and GPT-4 could, at the time of the study, not process these, we further investigated the potential image-related discrepancy between the examinations by excluding from subsequent analyses any questions that required image-dependent responses. The exclusion of these questions did not significantly alter examination difficulty, as evidenced by similar student scores (Figure 2D).

Moreover, no differences were observed in the parameters collected on student accuracy, questions, or answer characteristics in relation to the performance of GPT-4 and GPT-3.5 in the excluded cases (Multimedia Appendix 3). Upon excluding image-based questions, GPT-4 continued to outperform GPT-3.5, with scores approaching 91.44%. However, GPT-3.5 exceeded expectations by achieving passing scores on all 3 examinations (October 2021: 60.22%; April 2022: 63.36%; October 2022: 60.07%; Figure 2E and Multimedia Appendix 4). GPT-3.5’s accuracy (*P*=.66), the number of images (*P*=.07), and students’ accuracy (*P*=.77) remained constant throughout the examinations, whereas GPT-4’s accuracy (*P*=.02), the specialties (*P*<.001), and question type (*P*=.04) varied (Multimedia Appendix 4 and Figures 2A, 2B, and 2E). The details of the included questions and their respective categorizations are provided in Table 1.

**Table 1:** Summary statistics for ChatGPT's (GPT-3.5 and GPT-4) accuracy during the written German medical licensing examination, 2021-2022.

Characteristic		Overall (N=834)	Accuracy of GPT-3.5					Accuracy of GPT-4				
			False (n=323)	True (n=511)	*P* value		False (n=105)		True (n=729)		*P* value	
Students' correct response rate (%), mean (SD)		77 (18)	71 (18)	80 (16)	<.001^a^		70 (18)		78 (17)		<.001^a^	
Accuracy of GPT-3.5, n (%)		511 (61)	N/A^b^	N/A	N/A		38 (36)		473 (65)		<.001^c^	
Accuracy of GPT-4, n (%)		729 (87)	256 (79)	473 (93)	<.001^c^		N/A		N/A		N/A	
Readability score of the question, mean (SD)		14.96 (1.89)	14.93 (1.87)	14.98 (1.90)	.65^a^		14.91 (2.26)		14.97 (1.84)		.21^a^	
**Question type** **, n (%)**						.76^c^		N/A		N/A		.009^c^
	Connected (key feature)	532 (64)	204 (63)	328 (64)			79 (75)		453 (62)			
	Single question	302 (36)	119 (37)	183 (36)			26 (25)		276 (38)			
	Images referenced in questions	84 (10)	23 (7.1)	61 (12)	.02^c^		17 (16)		67 (9.2)		.03^c^	
**Specialty, n (%)**						.02^c^		N/A		N/A		.07^c^
	Gynecology	43 (5.2)	12 (3.7)	31 (6.1)			7 (6.7)		36 (4.9)			
	Infectiology	74 (8.9)	24 (7.4)	50 (9.8)			6 (5.7)		68 (9.3)			
	Internal medicine	176 (21)	71 (22)	105 (21)			15 (14)		161 (22)			
	Neurology	112 (13)	51 (16)	61 (12)			12 (11)		100 (14)			
	Others	269 (32)	106 (33)	163 (32)			46 (44)		223 (31)			
	Pediatrics	62 (7.4)	26 (8.0)	36 (7.0)			11 (10)		51 (7.0)			
	Psychiatry	54 (6.5)	11 (3.4)	43 (8.4)			5 (4.8)		49 (6.7)			
	Surgery	44 (5.3)	22 (6.8)	22 (4.3)			3 (2.9)		41 (5.6)			
**Expertise, n (%)**						.64^c^		N/A		N/A		.34^c^
	Background knowledge	103 (12)	32 (9.9)	71 (14)			13 (12)		90 (12)			
	Complications	49 (5.9)	19 (5.9)	30 (5.9)			4 (3.8)		45 (6.2)			
	Diagnostic competence	466 (56)	184 (57)	282 (55)			54 (51)		412 (57)			
	Prevention competence	36 (4.3)	13 (4.0)	23 (4.5)			6 (5.7)		30 (4.1)			
	Scientific practice	34 (4.1)	14 (4.3)	20 (3.9)			8 (7.6)		26 (3.6)			
	Therapeutic competence	146 (18)	61 (19)	85 (17)			20 (19)		126 (17)			

^a^Wilcoxon rank-sum test.

^b^N/A: not applicable.

^c^Pearson chi-square test.

**Figure 3:**
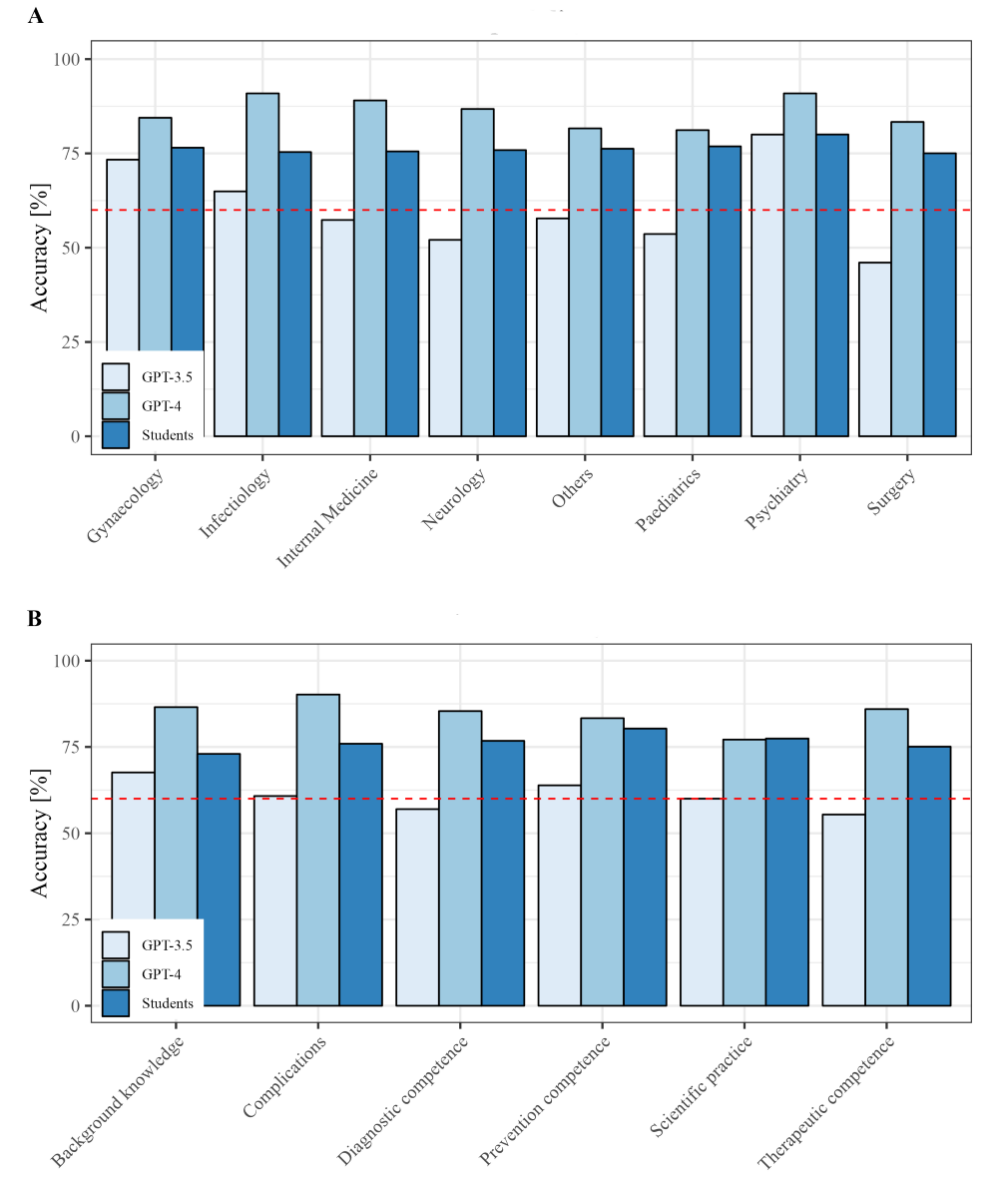
Comparison of ChatGPT's (GPT-3.5 and GPT-4) and students’ relative accuracy in relation to the tested specialties and methodology in the written German medical licensing examination (2021-2022). The bar graph displays the percentage of correct answers provided by ChatGPT (GPT-3.5 and GPT-4) and students in (A) each specialty and (B) and methodology, while the blue line demonstrates a 60% pass mark.

After controlling for all other variables, correct student responses (GPT-3.5: OR 0.01, 95% CI 0.00-0.01, *P*<.001; GPT-4: OR 0.00, 95% CI 0.00-0.00, *P*=.003) and questions with images (GPT-3.5: OR 0.19, 95% CI 0.08-0.30, *P*<.001; GPT-4: OR –0.09, 95% CI –0.16 to –0.01, *P*=.02) emerged as significant predictors of GPT-3.5’s and GPT-4’s accuracy, regardless of the version. Furthermore, our analysis revealed that only questions pertaining to psychiatry were significant predictors of correct GPT-3.5 responses (OR 0.19, 95% CI 0.02-0.36, *P*=.03). In contrast, questions related to internal medicine (OR 0.10, 95% CI 0.00-0.19, *P*=.04) and surgery (OR 0.12, 95% CI 0.00-0.25, *P*=.049) were the only medical subspecialties significantly predicting accurate responses of GPT-4. Conversely, questions concerning scientific practice (OR –0.14, 95% CI –0.29 to 0.00, *P*=.05) were less likely to be answered correctly by GPT-4 (Table 2 and Figure 3). The question SMOG readability score, however, did not significantly impact ChatGPT’s accuracy.

**Table 2:** Regression analysis to compare ChatGPT's (GPT-3.5 and GPT-4) accuracy during the written German medical licensing examination (2021-2022; N=833).

Characteristic	GPT-3.5									GPT-4						
	Univariate				Multivariate				Univariate					Multivariate		
	Odds ratio	95% CI	*P* value	β		95% CI	*P* value	Odds ratio			95% CI	*P* value	β		95% CI	*P* value
Students’ correct response rate	1.03	1.02 to 1.04	<.001	.01		0.00 to 0.01	<.001	1.02			1.01 to 1.03	<.001	.00		0.00 to 0.00	.003
Accuracy of GPT-4	3.25	2.13 to 5.02	<.001	.26		0.16 to 0.36	<.001	N/A^a^			N/A	N/A	N/A		N/A	N/A
Accuracy of GPT-3.5	N/A	N/A	N/A	N/A		N/A	N/A	3.25			2.13 to 5.02	<.001	.12		0.08 to 0.17	<.001
October 2021 examination	0.94	0.70 to 1.27	.68	.00		–0.08 to 0.08	.94	0.90			0.59 to 1.40	.64	.02		–0.04 to 0.07	.55
April 2022 examination	1.15	0.86 to 1.54	.35	.03		–0.05 to 0.11	.47	1.85			1.17 to 3.03	.01	.06		0.01 to 0.11	.03
October 2022 examination	0.92	0.69 to 1.24	.59	N/A		N/A	N/A	0.63			0.42 to 0.96	.03	N/A		N/A	N/A
Question type	0.96	0.72 to 1.28	.78	–.03		–0.10 to 0.04	.39	1.86			1.18 to 3.01	.01	.06		0.02 to 0.11	.007
Images referenced in questions	1.77	1.09 to 2.98	.03	.19		0.08 to 0.30	<.001	0.52			0.30 to 0.96	.03	–.09		–0.16 to –0.01	.02
Other specialty	0.96	0.71 to 1.30	.80	.00		–0.13 to 0.14	.94	0.57			0.37 to 0.86	.007	.02		–0.07 to 0.11	.73
Gynecology and obstetrics	1.62	0.84 to 3.33	.17	.12		–0.06 to 0.31	.19	0.71			0.32 to 1.78	.42	.01		–0.12 to 0.14	.88
Surgery	0.62	0.33 to 1.14	.12	–.12		–0.30 to 0.06	.18	2.03			0.72 to 8.49	.24	.12		0.00 to 0.25	.049
Internal medicine	0.92	0.66 to 1.30	.63	–.02		–0.15 to 0.12	.81	1.7			0.99 to 3.14	.07	.10		0.00 to 0.19	.043
Infectious diseases	1.35	0.82 to 2.28	.24	.06		–0.10 to 0.22	.48	1.7			0.78 to 4.48	.23	.09		–0.02 to 0.20	.11
Psychiatry	2.61	1.37 to 5.40	.005	.19		0.02 to 0.36	.03	1.44			0.62 to 4.23	.45	.03		–0.09 to 0.15	.61
Neurology	0.72	0.49 to 1.08	.12	–.04		–0.18 to 0.11	.61	1.23			0.68 to 2.45	.52	.08		–0.02 to 0.18	.11
Pediatrics	0.87	0.52 to 1.48	.60	N/A		N/A	N/A	0.64			0.34 to 1.34	.21	N/A		N/A	N/A
Diagnostic competence	0.93	0.70 to 1.23	.60	–.03		–0.17 to 0.11	.67	1.22			0.81 to 1.85	.33	–.05		–0.14 to 0.05	.34
Therapeutic competence	0.86	0.60 to 1.24	.41	–.04		–0.19 to 0.12	.65	0.89			0.54 to 1.54	.66	–.06		–0.16 to 0.05	.28
Background knowledge	1.47	0.95 to 2.32	.09	.08		–0.09 to 0.24	.36	1.00			0.55 to 1.94	>.99	–.05		–0.16 to 0.06	.36
Prevention competence	1.13	0.57 to 2.32	.74	.00		–0.20 to 0.20	>.99	0.71			0.31 to 1.93	.45	–.11		–0.25 to 0.03	.11
Scientific practice	0.90	0.45 to 1.85	.77	.01		–0.20 to 0.22	.95	0.45			0.21 to 1.09	.06	–.14		–0.29 to 0.00	.05
Complications	1.00	0.56 to 1.84	>.99	N/A		N/A	N/A	1.66			0.66 to 5.61	.34	N/A		N/A	N/A
Readability score of the question	1.01	0.94 to 1.09	.70	.01		–0.01 to 0.03	.24	1.02			0.91 to 1.14	.76	.00		–.01 to 0.01	.98

^a^N/A: not applicable.

## Discussion

### Principal Findings

With the introduction of ChatGPT’s GPT-3.5 and GPT-4 iterations, the potential application for AI in research, patient care, and medical education is gaining recognition [2,8,40]. By improving the users’ experience and facilitating more efficient information retrieval, ChatGPT might even revolutionize the future of search engines and shift the focus of medical education from memorization to practical application [8,10,11].

Under this premise, the nearly passing scores of the freely available GPT-3.5 iteration, along with the exceptional scores of GPT-4, are highly relevant. Even with the varying scores of 51%-67% of GPT-3.5 across various input languages [13-15,41,42], both models consistently outperform most prominent general and domain-specific LLMs, such as InstructGPT (53%), GPT-3 (25%), and BioMedLM (50%) [14,43,44]. Despite these improvements, GPT-3.5’s or GPT-4’s performance still fell short in comparison to that of medical students in a Japanese medical examination according to the study by Takagi et al [15]. In comparison to the German medical students, however, GPT-3.5 scored in the 8.6th percentile, while GPT-4 ranked in the 92.8th, 99.5th, and 92.6th percentiles in the October 2021, April 2022, and October 2022 examinations [39,45,46]. The observed variations in the AI's accuracy across input languages may partially reflect the language composition of their data sets, as LLMs tend to favor languages that are more represented in their training data [16,17]. Since ChatGPT appears to perform optimally with English inputs, language emerges as a limiting factor for its accuracy, suggesting that globally consistent application is dependent upon users' proficiency in English.

Moreover, the nearly 30% performance increase from GPT-3.5 to GPT-4, as indicated in this study and supported by a Japanese study, which suggests a similar language distribution within the GPT-3.5 and GPT-4 data sets [15]. GPT-4, unlike GPT-3.5, also did not answer questions containing images on repetition, showing an improvement in the previously incorrect content produced by GPT-4’s predecessor [17].

Thus, health care professionals could potentially benefit, especially from GPT-4’s conclusive and often nonobvious insights to multiple-choice questions, as these users have the ability to verify crucial details [13,14,41]. For instance, there is potential for using GPT-3.5 and GPT-4 in a medical education tutoring environment, as evidenced by its successful application in anatomy [47]. However, when using either GPT-3.5 or GPT-4 for medical applications, its differing accuracy across specialties must also be taken into account [48]. GPT-3.5 initially displayed a high degree of accuracy within the field of psychiatry, while GPT-4 demonstrated its strength in internal medicine and surgery. Considering the rising prevalence of psychiatric disorders and concomitant challenges in providing care, it seemed likely that nonprofessionals would also turn to the chatbot for mental health issues at the time of GPT-3.5’s release [8,49,50]. Hence, it is conceivable that GPT-3.5’s training data set includes not only a substantial and reliable portion of psychiatric data, but also its developers might have first fine-tuned ChatGPT specifically in this domain in anticipation of its high demand [51-53]. Thus, the developers might have also fine-tuned GPT-4 specifically in internal medicine and surgery, possibly reacting to a high demand in this area from users of its’ predecessor. GPT-4’s impressive performance is not limited to the medical field, as it demonstrated comparable percentile scores in the Uniform Bar Exam, showcasing it potential as a versatile tool across diverse academic disciplines [17]. However, assessing the possible reasons for the performance differences between GPT-3.5 and GPT-4 is complicated by the confidential architecture of GPT-4 [54], posing challenges for research on future applications.

In turn, GPT-4’s excellent achievements shed light on the limitations of current testing paradigms in medical education that often favor rote memorization over a critical and context-aware approach. They also highlight the inadequacy of multiple-choice questions as a means of assessing medical knowledge, as they tend to encourage binary thinking as “true” and “false,” which often fails to capture the complex reality of the medical practice [11]. Although GPT-3.5 and GPT-4 allow the simple and fast retrieval of medical information from any internet-capable device that fits in one's pocket [9,10], neither GPT-3.5 nor GPT-4 verifies the information they provide. Thus, ChatGPT's output needs to be approached with a critical mindset, recognizing that misinformation may be more difficult to detect than in the output of other search engines that offer multiple sources in response to a query and take login credentials into account [8,55]. To navigate these changing informational landscapes, a basic understanding in data science seems necessary alongside traditional medical expertise [56]. It may even be beneficial for future iterations of AI tools to include references to the sources underlying each search in order to increase transparency and allow users to assess the reliability of the information they receive.

In a previous study by Nov et al [57], considering that 59% of participants trusted chatbots more than traditional search engines, it must be noted that GPT-3.5 and GPT-4 have only been tested on medical examination questions and not questions by nonprofessionals, limiting general recommendations for unsupervised patient education or the general population. It seems evident that GPT-4 has been benchmarked against medical licensing examinations, explaining not only GPT-4’s excellent scores but also exceeding achievements in internal medicine and surgery, which, for instance, have been overrepresented in the medical examinations assessed in this study [12,17].

Since GPT-3.5 failed the German medical licensing examination by a narrow margin, its use for answering medical questions is generally not advisable. Moreover, the remarkable performance of GPT-4 in the German Medical State Examination may not be universally applicable outside a medical examination setting, especially considering that GPT-4 was presumably benchmarked on academic and professional examinations [17].

As literature on ChatGPT is scarce, and it can be difficult to detect incorrect output from this AI tool, the content it generates must be carefully assessed. Nevertheless, medical professionals may still be able to benefit from GPT-3.5’s and GPT-4’s explanations and, in some cases, gain new nonobvious insights. With the release of GPT-4’s ability to handle pictures on the horizon, the potential for further applications of GPT-3.5 and GPT-4 to improve the medical workflow or medical education seems eminent, emphasizing the need for continued research into AI.

### Limitations

This study’s findings on GPT-3.5’s and GPT-4’s medical proficiencies are limited to multiple-choice questions from the German medical licensing examination, which may not be representative of other types of examinations or contexts. However, it is worth noting that GPT-3.5 and GPT-4 have demonstrated similar performances in examinations in other countries and languages, which suggests some degree of generalizability.

In addition, the sample size of 937 questions and the exclusion of image-based questions may not capture the full range of difficulty levels or content areas. Although the collected parameters did not differ in terms of GPT-3.5’s and GPT-4’s accuracy in the excluded cases, the decision to exclude image-based questions may have introduced a sampling bias. By testing for differences, efforts were made to minimize this bias and maintain the integrity of the results.

As GPT-3.5’s and GPT-4’s performances were compared to those of German medical students using the MEDI-LEARN service, a selection bias might have been introduced. However, the high correlation between the MEDI-LEARN statistics and the IMPP statistics indicates at best a weak expression of this selection bias [58].

It should also be noted that a replication of this study might not yield the exact same results, as the literature suggests that GPT-3.5 is inconsistent in answering 15% of medical questions [59]. However, the trends observed in this study appear to be consistent with those reported in other published and preprint studies on GPT-3.5’s and GPT-4’s performance.

### Conclusions

In conclusion, the results of this study indicate that only GPT-4 consistently passed all 3 medical examinations, ranking in the 92.8th to 99.5th percentile in comparison to medical students. These findings highlight the strengths and limitations of ChatGPT in the context of medical examinations and raise questions about the future of medical education.

Although GPT-3.5’s and GPT-4’s accuracy in medical examinations seems consistent across different countries and languages, its inconsistencies, potential biases, and number of incorrect answers restrain a recommendation for its use by the general population for medical purposes. However, its elaborate explanations and potential to yield nonobvious insights may benefit medical professionals in training.

While this study hints to a moderate accuracy of GPT-3.5 and a stellar performance of GPT-4 in answering medical examination questions, further research is necessary to gain deeper insights, explore future applications, and ensure safe use of ChatGPT for end users.

## Appendix

Multimedia Appendix 1 Responses of (A) GPT-3.5 and (B) GPT-4 to the queries on its ability to pass a medical exam, 2023.

Multimedia Appendix 2 Summary statistics for all questions regarding exam time and ChatGPT’s (GPT-3.5 and GPT-4) accuracy in the German medical licensing exam, 2021-2022.

Multimedia Appendix 3 Summary statistics for excluded questions regarding ChatGPT’s (GPT-3.5 and GPT-4) accuracy in the German medical licensing exam, 2021-2022.

Multimedia Appendix 4 Summary statistics for included questions regarding exam time in the German medical licensing exam, 2021-2022.

## Abbreviations

AI: artificial intelligence
IMPP: Institut für Medizinische und Pharmazeutische Prüfungsfragen
LLM: large language model
SMOG: Simple Measure of Gobbledygook
