# Supplementary figures and images for: Comparison of the Performance of GPT-3.5 and GPT-4 With That of Medical Students on the Written German Medical Licensing Examination: Observational Study
Source: JMIR Med Educ. 2024 Feb 8;10:e50965. doi: 10.2196/50965 (PMC10884900; doi:10.2196/50965)

A


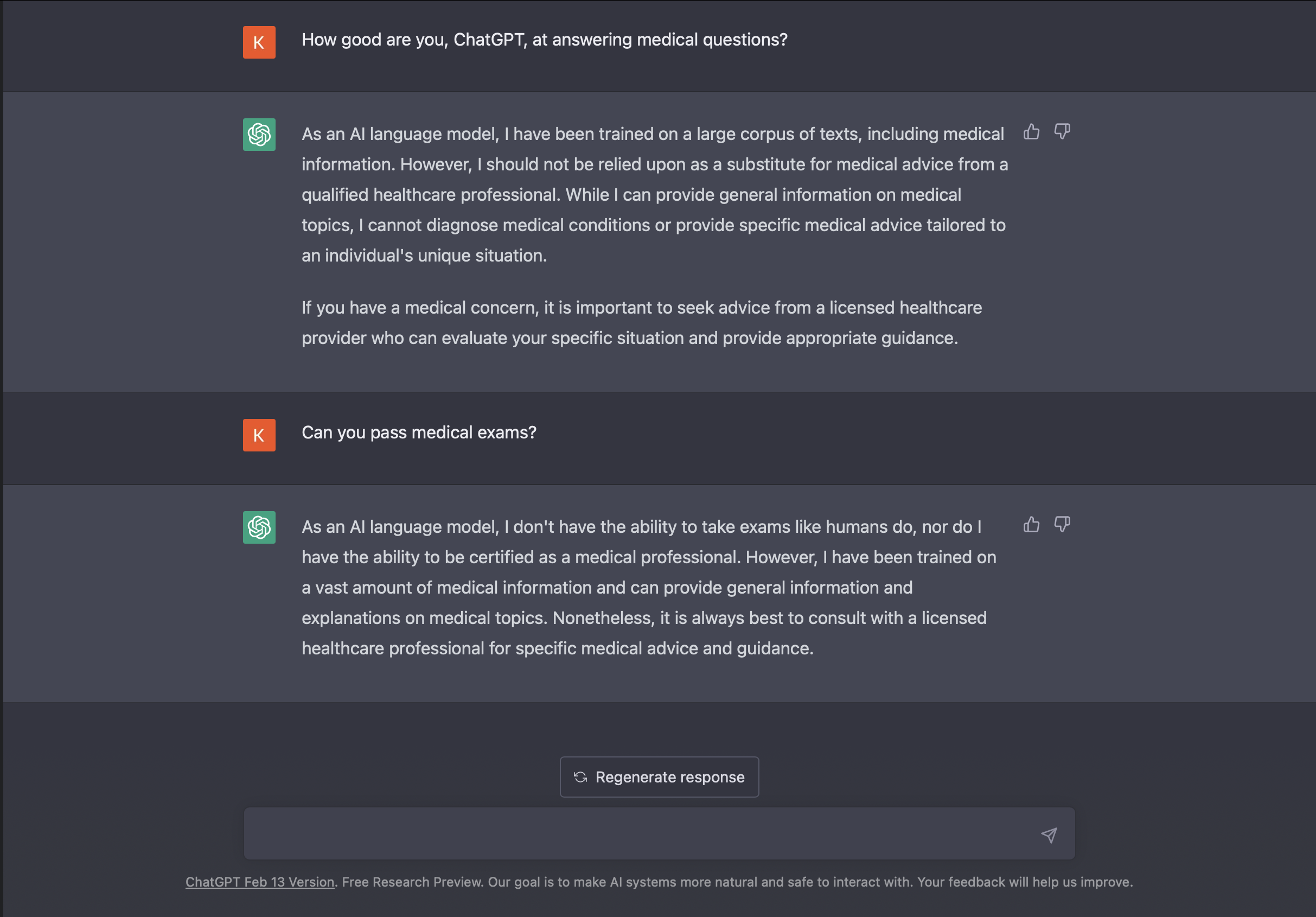


B


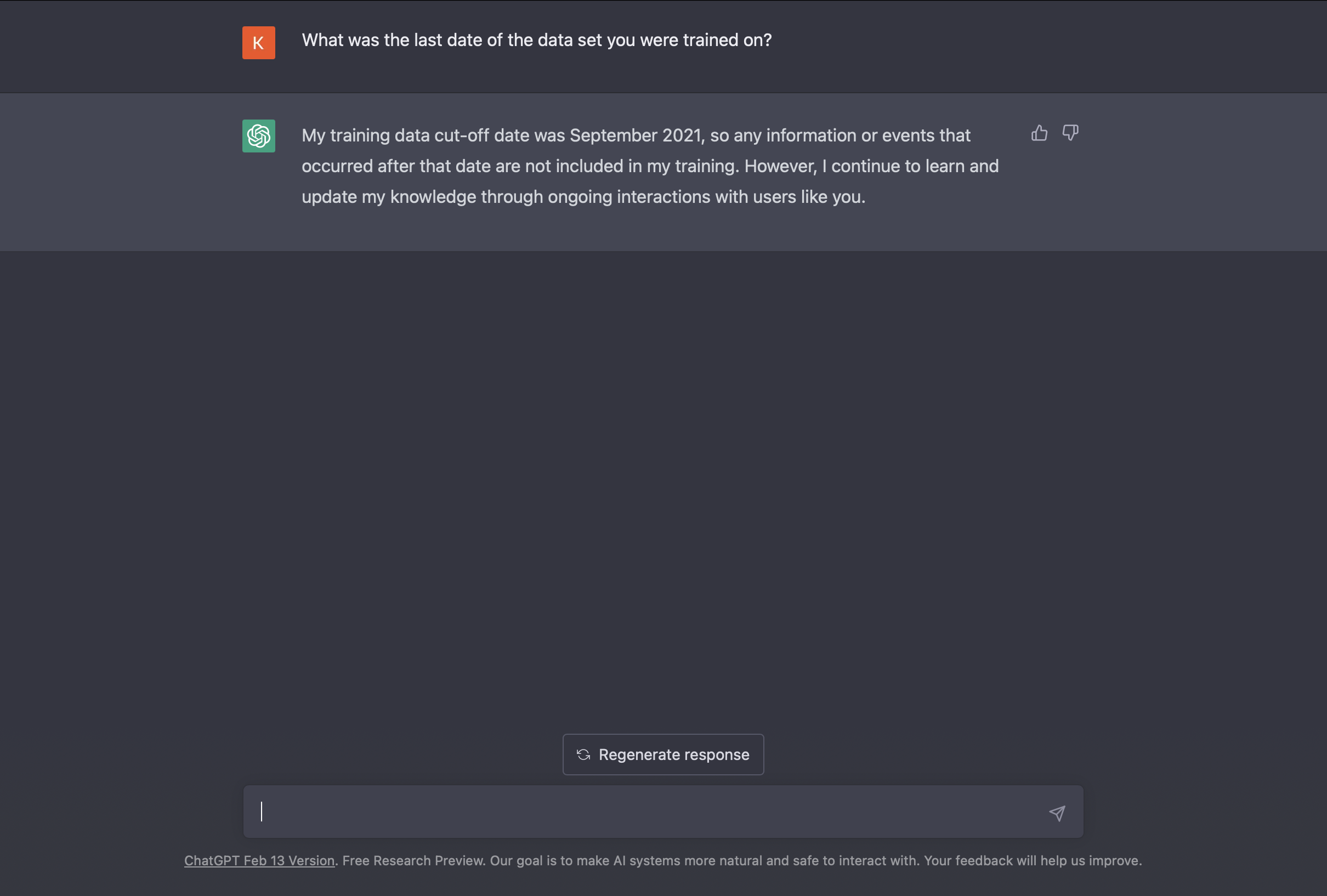

Supplement: Multimedia Appendix 1 [file mededu_v10i1e50965_app1.docx]
