# Supplementary material for: Comparison of the Performance of GPT-3.5 and GPT-4 With That of Medical Students on the Written German Medical Licensing Examination: Observational Study
Source: JMIR Med Educ. 2024 Feb 8;10:e50965. doi: 10.2196/50965 (PMC10884900; doi:10.2196/50965)
Supplement: Multimedia Appendix 2 [file mededu_v10i1e50965_app2.docx]

| **Characteristic** | **Time** | | | | **Accuracy GPT-3.5** | | | | **Accuracy GPT-4** | | | |
| --- | --- | --- | --- | --- | --- | --- | --- | --- | --- | --- | --- | --- |
|  | **April 2022**, N = 319 | **October 2021**, N = 307 | **October 2022**, N = 311 | **p-value** | **Overall**, N = 937 | **FALSE**, N = 389 | **TRUE**, N = 548 | **p-value** | **Overall**, N = 931 | **FALSE**, N = 135 | **TRUE**, N = 796 | **p-value** |
| **Students' correct response rate (%)** | 76 ± 18 | 76 ± 17 | 76 ± 18 | .86^c^ | 76 ± 18 | 71 ± 18 | 80 ± 17 | **<.001^d^** | 76 ± 18 | 70 ± 18 | 77 ± 17 | **<.001^d^** |
| **Accuracy of GPT-3.5 (N = 937).** | 195 / 319 (61%) | 180 / 307 (59%) | 173 / 311 (56%) | .37^b^ |  |  |  |  | 546 / 931 (59%) | 42 / 135 (31%) | 504 / 796 (63%) | **<.001^b^** |
| **Accuracy of GPT-4. (N = 931)** | 287 / 315 (91%) | 257 / 306 (84%) | 252 / 310 (81%) | **.002^b^** | 796 / 931 (85%) | 292 / 385 (76%) | 504 / 546 (92%) | **<.001^b^** |  |  |  |  |
| **Readibility score of question** | 15.01 ± 1.86 | 14.87 ± 1.97 | 14.91 ± 1.76 | .30^c^ | 14.93 ± 1.86 | 14.92 ± 1.84 | 14.93 ± 1.88 | .84**^d^** | 14.93 ± 1.86 | 14.83 ± 2.09 | 14.95 ± 1.82 | .12**^d^** |
| **Question type** |  |  |  | .057^b^ |  |  |  | .51^b^ |  |  |  | **.007^b^** |
| Connected (Key-Feature) | 189 / 319 (59%) | 206 / 307 (67%) | 209 / 311 (67%) |  | 604 / 937 (64%) | 246 / 389 (63%) | 358 / 548 (65%) |  | 601 / 931 (65%) | 101 / 135 (75%) | 500 / 796 (63%) |  |
| Single Question | 130 / 319 (41%) | 101 / 307 (33%) | 102 / 311 (33%) |  | 333 / 937 (36%) | 143 / 389 (37%) | 190 / 548 (35%) |  | 330 / 931 (35%) | 34 / 135 (25%) | 296 / 796 (37%) |  |
| **Included** | 292 / 319 (92%) | 269 / 307 (88%) | 273 / 311 (88%) | .21^b^ | 834 / 937 (89%) | 323 / 389 (83%) | 511 / 548 (93%) | **<.001^b^** | 834 / 931 (90%) | 105 / 135 (78%) | 729 / 796 (92%) | **<.001^b^** |
| **Images referenced in questions** | 47 / 319 (15%) | 68 / 307 (22%) | 71 / 311 (23%) | **.018^b^** | 186 / 937 (20%) | 89 / 389 (23%) | 97 / 548 (18%) | .05^b^ | 180 / 931 (19%) | 47 / 135 (35%) | 133 / 796 (17%) | **<.001^b^** |
| **Specialty** |  |  |  | **<.001^b^** |  |  |  | **.002^b^** |  |  |  | .17^b^ |
| Gynaecology | 15 / 319 (4.7%) | 13 / 307 (4.2%) | 17 / 311 (5.5%) |  | 45 / 937 (4.8%) | 12 / 389 (3.1%) | 33 / 548 (6.0%) |  | 45 / 931 (4.8%) | 7 / 135 (5.2%) | 38 / 796 (4.8%) |  |
| Infectiology | 26 / 319 (8.2%) | 23 / 307 (7.5%) | 28 / 311 (9.0%) |  | 77 / 937 (8.2%) | 27 / 389 (6.9%) | 50 / 548 (9.1%) |  | 77 / 931 (8.3%) | 7 / 135 (5.2%) | 70 / 796 (8.8%) |  |
| Internal Medicine | 69 / 319 (22%) | 92 / 307 (30%) | 50 / 311 (16%) |  | 211 / 937 (23%) | 90 / 389 (23%) | 121 / 548 (22%) |  | 210 / 931 (23%) | 23 / 135 (17%) | 187 / 796 (23%) |  |
| Neurology | 33 / 319 (10%) | 45 / 307 (15%) | 43 / 311 (14%) |  | 121 / 937 (13%) | 58 / 389 (15%) | 63 / 548 (11%) |  | 121 / 931 (13%) | 16 / 135 (12%) | 105 / 796 (13%) |  |
| Others | 97 / 319 (30%) | 90 / 307 (29%) | 109 / 311 (35%) |  | 296 / 937 (32%) | 125 / 389 (32%) | 171 / 548 (31%) |  | 294 / 931 (32%) | 54 / 135 (40%) | 240 / 796 (30%) |  |
| Paediatrics | 33 / 319 (10%) | 7 / 307 (2.3%) | 29 / 311 (9.3%) |  | 69 / 937 (7.4%) | 32 / 389 (8.2%) | 37 / 548 (6.8%) |  | 69 / 931 (7.4%) | 13 / 135 (9.6%) | 56 / 796 (7.0%) |  |
| Psychiatry | 24 / 319 (7.5%) | 22 / 307 (7.2%) | 9 / 311 (2.9%) |  | 55 / 937 (5.9%) | 11 / 389 (2.8%) | 44 / 548 (8.0%) |  | 55 / 931 (5.9%) | 5 / 135 (3.7%) | 50 / 796 (6.3%) |  |
| Surgery | 22 / 319 (6.9%) | 15 / 307 (4.9%) | 26 / 311 (8.4%) |  | 63 / 937 (6.7%) | 34 / 389 (8.7%) | 29 / 548 (5.3%) |  | 60 / 931 (6.4%) | 10 / 135 (7.4%) | 50 / 796 (6.3%) |  |
| **Expertise** |  |  |  | .76^b^ |  |  |  | .38^b^ |  |  |  | .68^b^ |
| Background knowledge | 34 / 319 (11%) | 31 / 307 (10%) | 40 / 311 (13%) |  | 105 / 937 (11%) | 34 / 389 (8.7%) | 71 / 548 (13%) |  | 104 / 931 (11%) | 14 / 135 (10%) | 90 / 796 (11%) |  |
| Complications | 15 / 319 (4.7%) | 17 / 307 (5.5%) | 19 / 311 (6.1%) |  | 51 / 937 (5.4%) | 20 / 389 (5.1%) | 31 / 548 (5.7%) |  | 51 / 931 (5.5%) | 5 / 135 (3.7%) | 46 / 796 (5.8%) |  |
| Diagnostic competence | 191 / 319 (60%) | 176 / 307 (57%) | 177 / 311 (57%) |  | 544 / 937 (58%) | 234 / 389 (60%) | 310 / 548 (57%) |  | 541 / 931 (58%) | 79 / 135 (59%) | 462 / 796 (58%) |  |
| Prevention competence | 13 / 319 (4.1%) | 9 / 307 (2.9%) | 14 / 311 (4.5%) |  | 36 / 937 (3.8%) | 13 / 389 (3.3%) | 23 / 548 (4.2%) |  | 36 / 931 (3.9%) | 6 / 135 (4.4%) | 30 / 796 (3.8%) |  |
| Scientific practice | 8 / 319 (2.5%) | 15 / 307 (4.9%) | 12 / 311 (3.9%) |  | 35 / 937 (3.7%) | 14 / 389 (3.6%) | 21 / 548 (3.8%) |  | 35 / 931 (3.8%) | 8 / 135 (5.9%) | 27 / 796 (3.4%) |  |
| Therapeutic competence | 58 / 319 (18%) | 59 / 307 (19%) | 49 / 311 (16%) |  | 166 / 937 (18%) | 74 / 389 (19%) | 92 / 548 (17%) |  | 164 / 931 (18%) | 23 / 135 (17%) | 141 / 796 (18%) |  |
| *a* Mean and std.-deviation or frequency (%)  *b* Pearson's Chi-squared test  *c* Kruskal-Wallis rank sum test  d Wilcoxon rank sum test | | | | | | | | | | | | |
