# Supplementary material for: Comparison of the Performance of GPT-3.5 and GPT-4 With That of Medical Students on the Written German Medical Licensing Examination: Observational Study
Source: JMIR Med Educ. 2024 Feb 8;10:e50965. doi: 10.2196/50965 (PMC10884900; doi:10.2196/50965)
Supplement: Multimedia Appendix 3 [file mededu_v10i1e50965_app3.docx]

| **Characteristic** | **Accuracy GPT-3.5.** | | | | **Accuracy GPT-4.** | | | |
| --- | --- | --- | --- | --- | --- | --- | --- | --- |
|  | **Overall**,  N = 103^a^ | **FALSE**,  N = 66 ^a^ | **TRUE**,  N = 37 ^a^ | ***P-value*** | **Overall**,  N = 97 ^a^ | **FALSE**,  N = 30 ^a^ | **TRUE**,  N = 67 ^a^ | ***P-value*** |
| **Time** |  |  |  | .10^b^ |  |  |  | .10^b^ |
| April 2022 | 27 / 103 (26%) | 17 / 66 (26%) | 10 / 37 (27%) |  | 23 / 97 (24%) | 3 / 30 (10%) | 20 / 67 (30%) |  |
| October 2021 | 38 / 103 (37%) | 20 / 66 (30%) | 18 / 37 (49%) |  | 37 / 97 (38%) | 13 / 30 (43%) | 24 / 67 (36%) |  |
| October 2022 | 38 / 103 (37%) | 29 / 66 (44%) | 9 / 37 (24%) |  | 37 / 97 (38%) | 14 / 30 (47%) | 23 / 67 (34%) |  |
| **Students' correct response rate (%)** | 72 ± 18 | 70 ± 18 | 74 ± 18 | .32^d^ | 72 ± 18 | 70 ± 18 | 72 ± 18 | .54^d^ |
| **Readibility score of question** | 14.69 ± 1.58 | 14.91 ± 1.65 | 14.30 ± 1.37 | .15^d^ | 14.71 ± 1.60 | 14.57 ± 1.37 | 14.77 ± 1.70 | .57^d^ |
| **Question type** |  |  |  | .064^b^ |  |  |  | .75^b^ |
| Connected (Key-Feature) | 72 / 103 (70%) | 42 / 66 (64%) | 30 / 37 (81%) |  | 69 / 97 (71%) | 22 / 30 (73%) | 47 / 67 (70%) |  |
| Single Question | 31 / 103 (30%) | 24 / 66 (36%) | 7 / 37 (19%) |  | 28 / 97 (29%) | 8 / 30 (27%) | 20 / 67 (30%) |  |
| **Images referenced in questions** | 102 / 103 (99%) | 66 / 66 (100%) | 36 / 37 (97%) | .36^c^ | 96 / 97 (99%) | 30 / 30 (100%) | 66 / 67 (99%) | >.99^c^ |
| **Specialty** |  |  |  | .16^c^ |  |  |  | .79^c^ |
| Gynaecology | 2 / 103 (1.9%) | 0 / 66 (0%) | 2 / 37 (5.4%) | .13^b^ | 2 / 97 (2.1%) | 0 / 30 (0%) | 2 / 67 (3.0%) | >.99^b^ |
| Surgery | 19 / 103 (18%) | 12 / 66 (18%) | 7 / 37 (19%) | .93^c^ | 16 / 97 (16%) | 7 / 30 (23%) | 9 / 67 (13%) | .25^c^ |
| Internal Medicine | 35 / 103 (34%) | 19 / 66 (29%) | 16 / 37 (43%) | .14^b^ | 34 / 97 (35%) | 8 / 30 (27%) | 26 / 67 (39%) | .25^b^ |
| Infectiology | 3 / 103 (2.9%) | 3 / 66 (4.5%) | 0 / 37 (0%) | .55^c^ | 3 / 97 (3.1%) | 1 / 30 (3.3%) | 2 / 67 (3.0%) | >.99^c^ |
| Psychiatry | 1 / 103 (1.0%) | 0 / 66 (0%) | 1 / 37 (2.7%) | .36^c^ | 1 / 97 (1.0%) | 0 / 30 (0%) | 1 / 67 (1.5%) | >.99^c^ |
| Neurology | 9 / 103 (8.7%) | 7 / 66 (11%) | 2 / 37 (5.4%) | .48^c^ | 9 / 97 (9.3%) | 4 / 30 (13%) | 5 / 67 (7.5%) | .45^c^ |
| Paediatrics | 7 / 103 (6.8%) | 6 / 66 (9.1%) | 1 / 37 (2.7%) | .42^c^ | 7 / 97 (7.2%) | 2 / 30 (6.7%) | 5 / 67 (7.5%) | >.99^c^ |
| Others | 27 / 103 (26%) | 19 / 66 (29%) | 8 / 37 (22%) | .43^c^ | 25 / 97 (26%) | 8 / 30 (27%) | 17 / 67 (25%) | .89^c^ |
| **Expertise** |  |  |  | .63^c^ |  |  |  | .24^c^ |
| Background knowledge | 2 / 103 (1.9%) | 2 / 66 (3.0%) | 0 / 37 (0%) | .53^c^ | 1 / 97 (1.0%) | 1 / 30 (3.3%) | 0 / 67 (0%) | .31^c^ |
| Complications | 2 / 103 (1.9%) | 1 / 66 (1.5%) | 1 / 37 (2.7%) | >.99^c^ | 2 / 97 (2.1%) | 1 / 30 (3.3%) | 1 / 67 (1.5%) | .53^c^ |
| Diagnostic competence | 78 / 103 (76%) | 50 / 66 (76%) | 28 / 37 (76%) | .97^b^ | 75 / 97 (77%) | 25 / 30 (83%) | 50 / 67 (75%) | .34^b^ |
| Prevention competence | 0 / 103 (0%) | 0 / 66 (0%) | 0 / 37 (0%) | >.99^c^ | 0 / 97 (0%) | 0 / 30 (0%) | 0 / 67 (0%) | >.99^c^ |
| Scientific practice | 1 / 103 (1.0%) | 0 / 66 (0%) | 1 / 37 (2.7%) | .36^c^ | 1 / 97 (1.0%) | 0 / 30 (0%) | 1 / 67 (1.5%) | >.99^c^ |
| Therapeutic competence | 20 / 103 (19%) | 13 / 66 (20%) | 7 / 37 (19%) | .89^b^ | 18 / 97 (19%) | 3 / 30 (10%) | 15 / 67 (22%) | .15^b^ |
| *^a^* Mean and std.-deviation or frequency (%)  *^b^* Pearson's Chi-squared test  *^c^* Fisher's exact test  *^d^* Wilcoxon rank sum test | | | | | | | | |
