# Supplementary material for: Comparison of the Performance of GPT-3.5 and GPT-4 With That of Medical Students on the Written German Medical Licensing Examination: Observational Study
Source: JMIR Med Educ. 2024 Feb 8;10:e50965. doi: 10.2196/50965 (PMC10884900; doi:10.2196/50965)
Supplement: Multimedia Appendix 4 [file mededu_v10i1e50965_app4.docx]

| **Characteristic** | **Overall**, N = 834^a^ | **Time** | | |  |
| --- | --- | --- | --- | --- | --- |
|  |  | **April 2022**, N = 292^a^ | **October 2021**, N = 269^a^ | **October 2022**, N = 273^a^ | **p-value** |
| **Students' correct response rate (%)** | 77 ± 18 | 76 ± 18 | 77 ± 17 | 77 ± 18 | .77^c^ |
| **Accuracy of GPT-3.5.** | 511 / 834 (61%) | 185 / 292 (63%) | 162 / 269 (60%) | 164 / 273 (60%) | .66^b^ |
| **Accuracy of GPT-4.** | 729 / 834 (87%) | 267 / 292 (91%) | 233 / 269 (87%) | 229 / 273 (84%) | **.023^b^** |
| **Readibility score of question** | 14.96 ± 1.89 | 14.99 ± 1.90 | 14.94 ± 2.01 | 14.95 ± 1.77 | .71^c^ |
| **Question type** |  |  |  |  | **.038^b^** |
| Connected (Key-Feature) | 532 / 834 (64%) | 172 / 292 (59%) | 171 / 269 (64%) | 189 / 273 (69%) |  |
| Single Question | 302 / 834 (36%) | 120 / 292 (41%) | 98 / 269 (36%) | 84 / 273 (31%) |  |
| **Images referenced in questions** | 84 / 834 (10%) | 20 / 292 (6.8%) | 30 / 269 (11%) | 34 / 273 (12%) | .067^b^ |
| **Specialty** |  |  |  |  | **<.001^b^** |
| Gynaecology | 43 / 834 (5.2%) | 13 / 292 (4.5%) | 13 / 269 (4.8%) | 17 / 273 (6.2%) |  |
| Infectiology | 74 / 834 (8.9%) | 25 / 292 (8.6%) | 22 / 269 (8.2%) | 27 / 273 (9.9%) |  |
| Internal Medicine | 176 / 834 (21%) | 60 / 292 (21%) | 73 / 269 (27%) | 43 / 273 (16%) |  |
| Neurology | 112 / 834 (13%) | 32 / 292 (11%) | 42 / 269 (16%) | 38 / 273 (14%) |  |
| Others | 269 / 834 (32%) | 89 / 292 (30%) | 82 / 269 (30%) | 98 / 273 (36%) |  |
| Paediatrics | 62 / 834 (7.4%) | 31 / 292 (11%) | 7 / 269 (2.6%) | 24 / 273 (8.8%) |  |
| Psychiatry | 54 / 834 (6.5%) | 24 / 292 (8.2%) | 22 / 269 (8.2%) | 8 / 273 (2.9%) |  |
| Surgery | 44 / 834 (5.3%) | 18 / 292 (6.2%) | 8 / 269 (3.0%) | 18 / 273 (6.6%) |  |
| **Expertise** |  |  |  |  | .61^b^ |
| Background knowledge | 103 / 834 (12%) | 33 / 292 (11%) | 30 / 269 (11%) | 40 / 273 (15%) |  |
| Complications | 49 / 834 (5.9%) | 15 / 292 (5.1%) | 16 / 269 (5.9%) | 18 / 273 (6.6%) |  |
| Diagnostic competence | 466 / 834 (56%) | 172 / 292 (59%) | 149 / 269 (55%) | 145 / 273 (53%) |  |
| Prevention competence | 36 / 834 (4.3%) | 13 / 292 (4.5%) | 9 / 269 (3.3%) | 14 / 273 (5.1%) |  |
| Scientific practice | 34 / 834 (4.1%) | 7 / 292 (2.4%) | 15 / 269 (5.6%) | 12 / 273 (4.4%) |  |
| Therapeutic competence | 146 / 834 (18%) | 52 / 292 (18%) | 50 / 269 (19%) | 44 / 273 (16%) |  |
| *^a^* Mean and std.-deviation or frequency (%)  *^b^* Pearson's Chi-squared test  *^c^*  Kruskal-Wallis rank sum test | | | | | |
